# Novel Metric for Non-Invasive Beat-to-Beat Blood Pressure Measurements Demonstrates Physiological Blood Pressure Fluctuations during Pregnancy

**DOI:** 10.3390/s24103151

**Published:** 2024-05-15

**Authors:** David Zimmermann, Hagen Malberg, Martin Schmidt

**Affiliations:** Institute of Biomedical Engineering, TU Dresden, Fetscherstr. 29, 01307 Dresden, Germany; david.zimmermann@tu-dresden.de (D.Z.);

**Keywords:** beat-to-beat blood pressure acquisition, non-invasive biosignals, blood pressure fluctuation, beat morphology, pregnancy, physiological behavior

## Abstract

Beat-to-beat (B2B) variability in biomedical signals has been shown to have high diagnostic power in the treatment of various cardiovascular and autonomic disorders. In recent years, new techniques and devices have been developed to enable non-invasive blood pressure (BP) measurements. In this work, we aim to establish the concept of two-dimensional signal warping, an approved method from ECG signal processing, for non-invasive continuous BP signals. To this end, we introduce a novel BP-specific beat annotation algorithm and a B2B-BP fluctuation (B2B-BPF) metric novel for BP measurements that considers the entire BP waveform. In addition to careful validation with synthetic data, we applied the generated analysis pipeline to non-invasive continuous BP signals of 44 healthy pregnant women (30.9 ± 5.7 years) between the 21st and 30th week of gestation (WOG). In line with established variability metrics, a significant increase (*p* < 0.05) in B2B-BPF can be observed with advancing WOGs. Our processing pipeline enables robust extraction of B2B-BPF, demonstrates the influence of various factors such as increasing WOG or exercise on blood pressure during pregnancy, and indicates the potential of novel non-invasive biosignal sensing techniques in diagnostics. The results represent B2B-BP changes in healthy pregnant women and allow for future comparison with those signals acquired from women with hypertensive disorders.

## 1. Introduction

Beat-to-beat (B2B) variations in biomedical signals, such as photoplethysmography (PPG), electrocardiography (ECG), and blood pressure (BP), reflect natural variability that occurs from one heartbeat to the next. This variability is an important indicator of underlying physiological processes and has significant value for the diagnosis and treatment of various cardiovascular and autonomic disorders. In ECG research, B2B analyses have revealed high diagnostic power, such as the recently presented detection of patients with paroxysmal atrial fibrillation while in sinus rhythm [1]. New techniques and devices were developed to extend the non-invasive accessible parameter scope, and therefore, non-invasive BP measurements became accessible [2,3,4]. Non-invasive signal acquisition methods include approaches based on the volume clamp method [5] as well as optical pulsatile blood flow measurement approaches such as fiber-based diffuse speckle contrast analysis (DSCA) with and without multimode detection fibers which allow for the indirect estimation of the BP-B2B waveform [6,7]. Linear and non-linear time series measures were developed and investigated to study relationships of variations in B2B-BP signals with physiological and pathophysiological processes [8]. These measures were deployed to determine the relationship between BP variability (BPV) and the risk of falls in fallers and non-fallers via the evaluation of standard deviation (SD), root mean square (RMS), average real variability (ARV), standard deviation of the real variability, and RMS of the real variability of the systolic values and revealed that reduced B2B-BPV while standing is independently associated with increased risk of falls [9]. In another study, the role of B2B-BPV in early vascular aging was accessed by the determination of the total arterial compliance in cold pressure tests via SD, coefficient of variance, variance independent of mean, ARV, stroke volume (SV), and relative standard deviation and showed an increase in all variability parameters in the cold stimulus phase [10]. Furthermore, obstructive sleep apnea was researched in [11] via the evaluation of the SD of systolic and diastolic BP in combination with ECG recordings and unveiled a representation of sex-specific autonomic dysfunction by B2B-BP changes. Many more B2B-BP research applications and findings exist; for a precise and comprehensive review, refer to [8]. However, the parameters used in B2B-BP research consider specific systolic and diastolic pressure values only. Recent research in ECG B2B analysis [12] demonstrated a gain in information when considering not only the variability of single points in time or amplitude but also the entire ECG waveform, and in a combined measure of time and amplitude, B2B beat morphology changes. Also, two-dimensional signal warping (2DSW) was shown to robustly extract variability information for each beat in ECG signals [13,14]. Focusing on the robust detection of subtle quasiperiodic changes as well as a generalized implementation, the method is feasible in various applications. 

**Figure 1:**
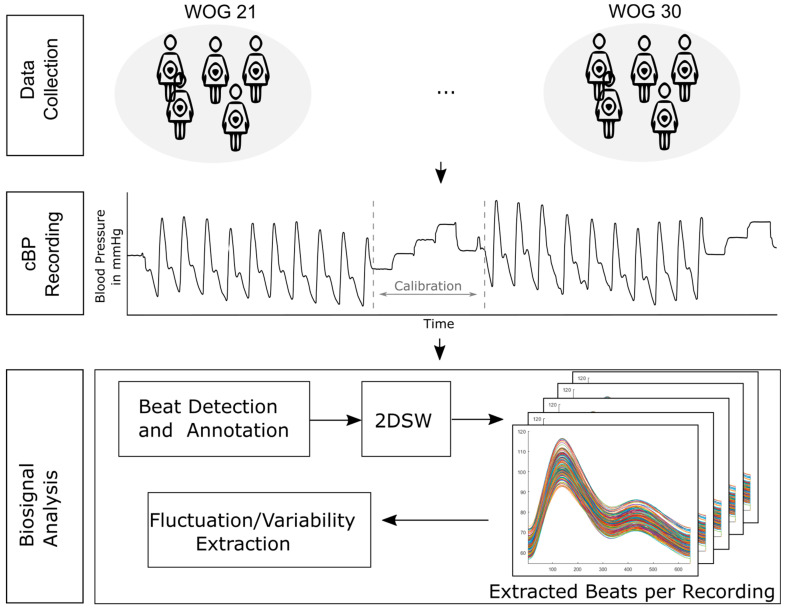
Overview of analysis steps. Pregnant women underwent repeated non-invasive cBP recordings between the 21st and 30th WOG. Within the raw signals, beats were detected and features of interest (FI, food index; SI, systolic index; NI, notch index; PI, peak index; and DI, diastolic index) were annotated. Each beat was processed with two-dimensional signal warping (2DSW), followed by fluctuation and variability extraction and statistical modeling.

To this end, this paper presents a novel approach to extracting B2B-BP information in time and amplitude from the entire BP waveform based on 2DSW. Therefore, we implemented a feature detector to annotate beats in non-invasive continuous BP signals (cBP), adapted the existing 2DSW algorithm to cBP signal analysis, and implemented a new B2B-BP fluctuation measure to consider changes in time and amplitude. After the validation of synthetic data to demonstrate the robustness and sensitivity of the adapted approach, we applied our processing pipeline to non-invasive cBP signals of healthy pregnant women who participated in the Fetal Autonomic Cardiovascular rEgulation (FACE) [15] study (Figure 1). In the FACE study, different biomedical signals, such as the BP and ECG of pregnant women, were recorded weekly between the 21st and 30th week of gestation (WOG). It is well-established that BP undergoes significant changes throughout pregnancy, with the first trimester showing a decrease [16] due to the relaxation of the vascular smooth muscle [17], while in the second trimester, it returns to pre-pregnancy levels. By the third trimester, BP begins to increase, reaching its highest levels just before delivery. These changes are essential for ensuring adequate placental perfusion and fetal growth [16]. Considering these pregnancy-induced effects on BP, we hypothesize changes in B2B-BP fluctuations between the different WOGs of the second and third trimesters. First, we wanted to determine if different WOGs have a significant impact on B2B-BP changes. As the volunteers per WOG group were not identical (Figure S1), potential impacts of women’s physiological attributes on B2B-BP changes between WOGs were considered. Second, our aim was to investigate the impact of both slow- and fast-breathing exercises on B2B-BP changes and to examine the influence of increasing WOG (WOG progression) on these alterations. Finally, to validate the results, we compared the numerical results to those in the literature. 

## 2. Materials and Methods

### 2.1. BP Beat Annotation with ELZA Algorithm

**Figure 2:**
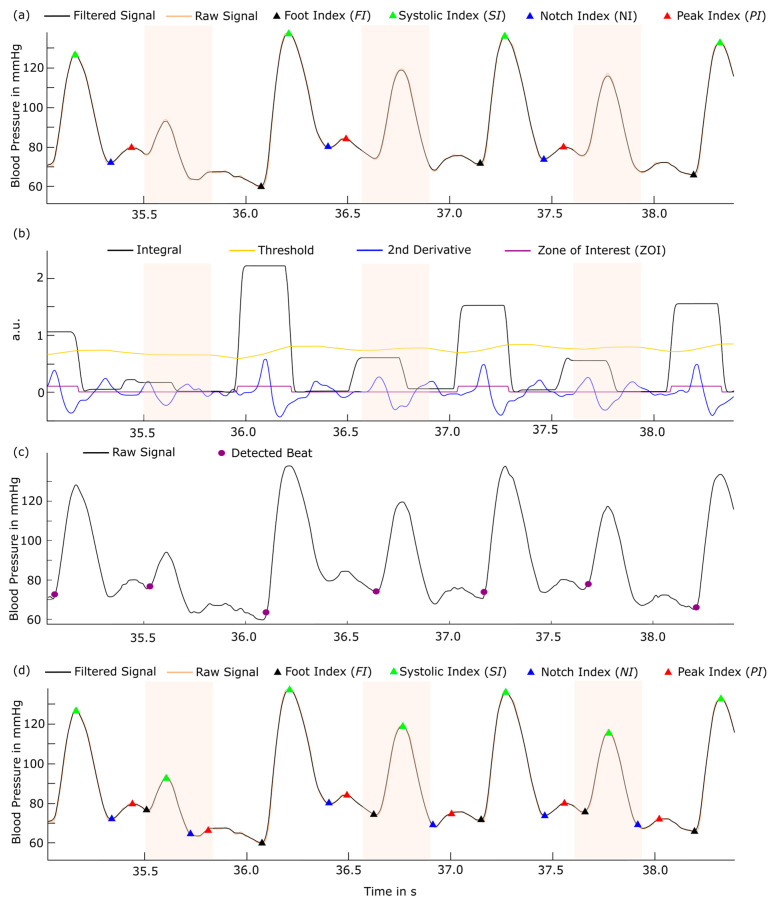
Blood pressure-specific beat detection and annotation (**a**,**b**) using the BPAA algorithm by Laurin [9] and (**c**) Zong et al. [19]. The advantages of both algorithms were combined in (**d**) the Extended-Laurin-Zong-Algorithm (ELZA). In (**a**), some beats (orange-coloured intervals) were not successfully detected and annotated since in (**b**), the integral (black) did not reach the threshold (yellow). In (**c**), the red circles indicate successful detection of all beats, but additional characteristic points (FI, SI, NI, PI and DI) are missing. Finally, in (**d**), the ELZA algorithm is able to identify and annotate all beats correctly and provides desired characteristic points. FI, food index; SI, systolic index; NI, notch index; PI, peak index; DI, diastolic index.

Several open-source detection algorithms in the literature provide the identification of characteristic points in B2B-BP signals. Such algorithms are the “BP-annotate” algorithm (BPAA) [18] as well as the WABP detector [19]. The BPAA algorithm identifies the beginning of a beat (foot index, FI), the maximum of the pressure signal (systolic maximum, SI), a dicrotic notch (notch index, NI), the dicrotic peak (peak index, PI), and the diastolic minimum (diastolic index, DI), as shown in Figure 2a. FI is identified within a zone of interest (ZOI), and SI represents the maximum peak after FI, as illustrated in Figure 2a,b. NI reflects the minimum between SI and DI, and PI is detected as the maximum in the second derivate. The ZOI is located between the intercepts of a defined threshold and the integral. In our signals, we found missing annotations using BPAA since the integral amplitude was below the threshold, as shown in Figure 2b (colored intervals). In contrast, the WABP algorithm identified all beats correctly but was lacking annotation of FI, SI, NI, PI, and DI points, as shown in Figure 2c. 

To merge the advantages of correctness and scope in detection, we combined both algorithms into an Extended-Laurin-Zong-Algorithm (ELZA), as shown in Figure 2d. BPAA is limited to 200 Hz by input filter constraints and downsample signals of higher frequency, which decreases detection precision. That is why ELZA was equipped with high-frequency signal processing by adding sampling frequency ($f_{s}$)-dependent finite impulse response filter (FIR) of the order of $f_{s}*40^{-1}$ with the passband b=0.2−2 Hz, followed by a moving average filter with the frequency-dependent window size $w(f_{s})=f_{s}*20^{-1}$. Signal shift is avoided by using the zero-phase filtering function. To prevent wrong physiological detections, we only allow beat detections with a minimal distance of 500 ms.

### 2.2. Functionality and Adaptation of i2DSW Algorithm

The iterative 2DSW (i2DSW) algorithm is able to identify time and amplitude B2B changes in quasiperiodic signals [13,14]. While initially implemented for B2B analysis of ECGs, the algorithm’s conception generally allows the adaptation to other quasiperiodic signals. The i2DSW algorithm is conceptionally based on the divide-and-conquer principle applied to continuous signal processing. Therefore, a template is initially generated from all the beats of a signal. This generated template is compared to each individual beat. Therefore, i2DSW performs minimization of the normalized Euclidean distance $d^{\left(2n\right)}(X^{y},\;Y^{y})$ between the template $Y^{y}$ and beat signal $X^{y}$. To achieve maximum minimization, the axes are separated into a grid yielding several areas. Each area is surrounded by four intercepts, called warping points $P_{i}$, which can be moved by shifting, thus deforming the warping grid and the corresponding signal. The mathematical details of this deformation can be found in [14]. To reach an optimal fit, the grid’s resolution is iteratively increased. With increasing iteration steps, smaller fragments of the template are warped and allow more robust adaptation to higher changes in signal morphology [16]. The minimization of $d^{\left(2n\right)}$ can be represented as a solution to the mathematical optimization problem of minimizing a cost function:

$$d^{\left(2n\right)}(X^{y},\;Y^{y})=\frac{\sqrt{\sum_{i=1}^{\left|X\right|}{\left(X^{y}(i)-Y^{y}(i)\right)}^{2}}}{\left|X\right|}\;$$

where |X| represents the length of X. To avoid overfitting, convergence criteria are implemented.

### 2.3. Parametrization

To adapt the i2DSW algorithm to B2B-BP signals, parameterization has been performed in parallel to [13]. Two convergence criteria are introduced to avoid overfitting of the optimization: the difference ${∆d}_{iter}^{\left(2n\right)}\;$ and the optimization relation ${\Phi d}_{iter}^{\left(2n\right)}$ between the current iteration (iter) and the following iteration (iter + 1). An iteration step is only applied if the following two conditions are true:

$${∆d}_{iter}^{\left(2n\right)}\leq d_{iter}^{\left(2n\right)}-d_{iter+1}^{\left(2n\right)}$$

$${\Phi d}_{iter}^{\left(2n\right)}\leq \left(1-\frac{d_{iter+1}^{\left(2n\right)}}{{\Phi d}_{iter}^{\left(2n\right)}}\right).$$

Based on the validation results, the following empirical determined convergence criteria were applied: (read: iteration 2 is not applied if absolute optimization ${∆d}_{iter}^{\left(2n\right)}\leq 0.02$ and relative optimization ${\Phi d}_{iter}^{\left(2n\right)}\leq 0.2$)

$${∆d}_{iter}^{\left(2n\right)}\leq \left\{\begin{array}{c} 0.020\;\;\;iter=2\\ 0.010\;\;\;iter=3\\ 0.005\;\;\;iter=4 \end{array}\right.$$

and

$${\Phi d}_{iter}^{\left(2n\right)}\leq \left\{\begin{array}{c} 0.20\;\;\;iter=2\\ 0.10\;\;\;iter=3\\ 0.05\;\;\;iter=4 \end{array}\right.$$

### 2.4. Calculation of Fluctuation Parameters

In order to fully exploit the possibilities of the i2DSW algorithm and to create an interpretable parameter that characterizes morphologic changes, we introduce fluctuation Γ in BP signals by taking changes along the entire waveform into account. Γ was shown to yield new insights into the characterization of ventricular repolarization in ECG signals compared to existing measures [12]. Briefly, Γ can be calculated in time $\Gamma_{x}$ and amplitude $\Gamma_{y}$. The generated data matrix A compromises warped N positions of M beats of the selected axis. Here, $x_{m,1}$ represents the foot index (FI) of each beat *m*, and $x_{m,N}$ represents the diastolic index (DI). Based on A, a median beat signal is calculated and the median coordinate $\tilde{x}$ is subtracted from each beat m and coordinate n.

$$A=\left(\begin{array}{ccc} \begin{array}{cc} x_{1,1}-{\tilde{x}}_{1} & x_{1,2}-{\tilde{x}}_{2}\\ x_{2,1}-{\tilde{x}}_{1} & x_{2,2}-{\tilde{x}}_{2} \end{array} & \cdots & \begin{array}{c} x_{1,N}-{\tilde{x}}_{N}\\ x_{2,N}-{\tilde{x}}_{N} \end{array}\\ \vdots & \ddots & \vdots \\ \begin{array}{cc} x_{M,1}-{\tilde{x}}_{1} & x_{M,2}-{\tilde{x}}_{2} \end{array} & \cdots & x_{M,N}-{\tilde{x}}_{N} \end{array}\right)$$

From this median-free matrix A, the variability of each individual coordinate within all the beats is identified by calculating its standard deviation $\sigma_{x,n}$ based on the mean ${\tilde{X}}_{n}$. This results in a fluctuation matrix ($F_{x}$) containing $\sigma_{x,n}$ for each coordinate:

$$\sigma_{x,n}=\sqrt{\frac{1}{M-1}\sum_{i=1}^{M}{\left(X_{i,n}-{\tilde{X}}_{n}\right)}^{2}}$$

$$F_{x}={(\sigma }_{x,1}\;\;\;\sigma_{x,2}\cdots \sigma_{x,N})$$

Finally, $\Gamma_{x}$ is calculated as the median of the matrix $F_{x}$

$$\Gamma_{x}={\tilde{F}}_{x}=\left\{\begin{array}{l} \sigma_{x,\frac{N+1}{2}}\;\;\;\;\;\;\;\;\;\;\;\;\;\;\;\;\;\;\;\;\;\;for\;N\;even\\ \frac{1}{2}(\sigma_{x,\frac{N}{2}}+\sigma_{x,\frac{N}{2}+1})\;for\;N\;odd \end{array}\right.$$

and in parallel, $\Gamma_{y}$ is calculated as the median of the matrix $F_{y}$.

$$\Gamma_{y}={\tilde{F}}_{y}=\left\{\begin{array}{l} \sigma_{y,\frac{N+1}{2}}\;\;\;\;\;\;\;\;\;\;\;\;\;\;\;\;\;\;\;\;\;\;for\;N\;even\\ \frac{1}{2}(\sigma_{y,\frac{N}{2}}+\sigma_{y,\frac{N}{2}+1})\;for\;N\;odd \end{array}\right.$$

### 2.5. Validation

#### 2.5.1. Simulated Data

**Figure 3:**
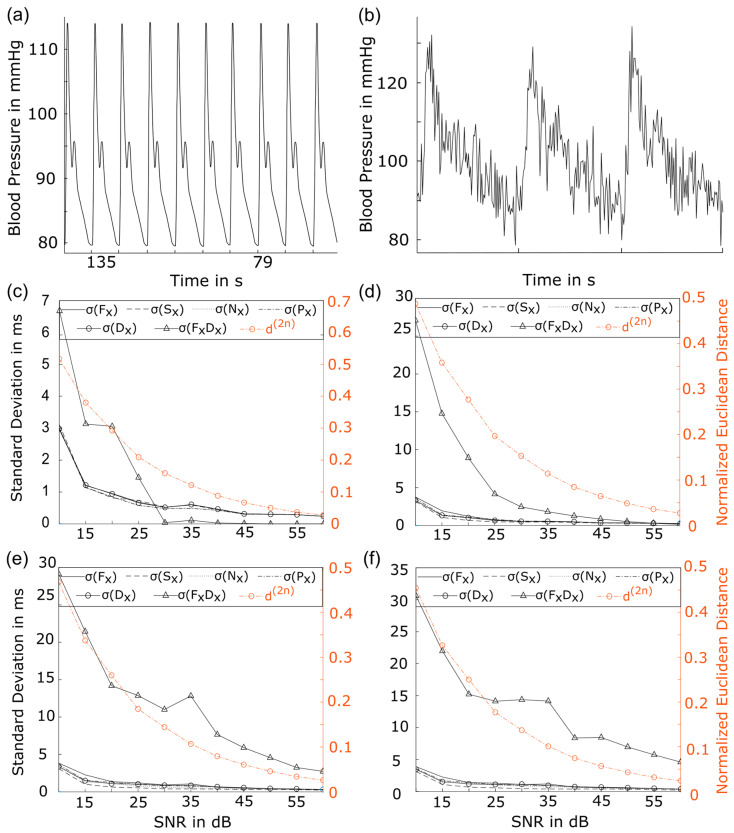
Validation of i2DSW on B2B-BPV. In (**a**), a synthetic optimal beat-to-beat blood pressure signal without any differences between the beats, different interferences such as (**b**) white gaussian noise with an SNR of 30 dB were added. i2DSW was performed, and for each iteration standard deviation σ of the x-coordinates between detected characteristic points $p\in \left\{FI,\;SI,\;NI,\;PI,DI\right\}$, $F_{x}D_{x}$ and Euclidean distance $d^{\left(2n\right)}$ was investigated, as shown in (**c**–**f**). FI, food index; SI, systolic index; NI, notch index; PI, peak index; DI, diastolic index; σ, standard deviation (SD); $d^{\left(2n\right)}$, euclidean distance.

To show the robustness of the parameterized i2DSW algorithm using ELZA for blood pressure fluctuation determination, we generated synthetic data by concatenating a noise-free beat 100 times. The ideal synthetic blood pressure signal without physiological variance, shown in Figure 3a, was created to identify detection and warping results and avoid the misinterpretation of technical variance as physiological variance. Different intensities of Gaussian white noise were added to the synthetic signal, resulting in signals with different signal-to-noise ratios (SNRs). Figure 3b shows the cutout of a representative signal with the SNR = 30 dB. To validate the warping results for all M beats, standard deviations σ of shifted x positions of the detected characteristic points $F_{x},S_{x},N_{x},P_{x},\;and\;D_{x}$ were compared to $d^{\left(2n\right)}$. Since the fluctuation measure Γ between $F_{x}$ and $D_{x}$ are used in later analyses, we also checked for the standard deviation of $F_{x}D_{x}=D_{x,m}-F_{x,m}$.

#### 2.5.2. Validation Results

Figure 3c–f shows the validation results of warping iterations 1, 2, 3, and 4. The overfitting criteria were disabled for demonstration purposes. The abscissa shows synthetic signals arranged from low SNR (low signal quality) to high SNR (high noise level) values. The left ordinate (blue) qualifies σ values, and the right ordinate qualifies Euclidean distance $d^{\left(2n\right)}$ (orange). Comparing all four iterations, a continuous decrease in all σ measures as well as in the $d^{\left(2n\right)}$ measure can be observed in relation to increasing SNR values. No difference in minimal $d^{\left(2n\right)}$ is seen between the different iterations. In contrast, SD values increase with a higher iteration cycle, e.g., in iteration 1, $\sigma \left(F_{x}\left(10dB\right)\right)=3\;ms$, and in iteration 4, $\sigma \left(F_{x}\left(10dB\right)\right)=4.7\;ms$. Similarly, in iteration 1, $\sigma \left(F_{x}D_{x}\left(10dB\right)\right)=6.8\;ms$, and in iteration 4, $\sigma \left(F_{x}D_{x}\left(10dB\right)\right)=30.7\;ms$. This characteristic was already described as an overfitting characteristic. The convergence criteria we have defined prevent i2DSW from entering the next iteration.

### 2.6. Study Design

B2B-BP changes during pregnancy were studied in continuous blood pressure data from the Fetal Autonomic Cardiovascular rEgulation (FACE) study [15]. The FACE study provided week-to-week datasets of 44 healthy pregnant women between the 21st and 30th week of gestation (WOG). The mean age of the volunteers was 30.9 ± 5.7 years, and the data were collected at the University of Leipzig Medical Center in cooperation with the TU Dresden and the Humboldt University Berlin. One aim of this study was to characterize the reaction of fetal autonomic regulation to maternal paced breathing based on a context-dependent biosignal analysis of ECG, PPG, BP, and other recorded signals. The participants underwent various exercises, including resting, paced breathing, and orthostasis. The data were acquired in the supine position. The intervention consisted of 5 min of slow breathing (7–8 respiration cycles per minute), 5 min of fast breathing (20 respiration cycles per minute), and a change in position from supine to orthostatic and back (Table 1b). During this study, a 5-min resting interval was performed between interventions. Table 1a and Figure S1 show the anthropometric characteristics of the women, including age, weight, and height. A total of 241 datasets were evaluated because not every volunteer participated in every WOG. To study the relationship between B2B-BP changes and pregnancy progression with reasonable statistical power, the datasets were merged, according to WOG, into four groups of similar size, namely WOG 21–23 (65 datasets), WOG 24–25 (64 datasets), WOG 26–27 (59 datasets), and WOG 28–30 (53 datasets). 

### 2.7. Non-Invasive B2B-BP Signal Measurement 

The BP signals were recorded by ambulatory BP monitoring (ABPM) using a PortaPres device (Finapres Medical Systems; Enschede, The Netherlands). This device applies the volume clamp method [5] by incorporating an inflatable cuff (attached to the middle finger) with an integrated plethysmograph. In an initial calibration, the unloaded finger artery diameter is determined by establishing the equilibrium between finger cuff pressure and intra-arterial pressure. During measurement, the plethysmograph detects the diameter of the finger arteries throughout blood pressure pulse cycles and feedbacks this measure to the inflatable cuff controller, which aims to keep the pressure of the artery unchanged at the unloading state. The required pressure to maintain this equilibrium resamples the arterial blood pressure in the finger. For brachial arterial pressure calibration, an additional arm cuff is used [20]. 

To maintain accuracy, the equilibrium pressure is constantly re-determined, which is represented by the calibration interval in the signal shown in Figure 1. For further signal processing, the recorded raw data were transferred from the ABPM to a standard computer. 

### 2.8. Signal Processing and B2B-BP Assessment

BB-BP raw data were annotated with the introduced ELZA algorithm (see Section 2.1). The calibration intervals were excluded in further analysis. The i2DSW algorithm was applied with a maximum of four iterations and the introduced convergence criteria (see Section 2.2 and Section 2.3). Considering the literature [9], B2B-BP variability parameters (B2B-BPVs), i.e., standard deviation (SD) and average real variability (ARV), were calculated based on the warped systolic indexes (SI) and diastolic indexes (DI), which resulted in parameters SBP-SD, DBP-SD, SBP-ARV, and DBP-ARV. B2B-BP fluctuation parameters (B2B-BPFs), i.e., $\Gamma_{x}$, $\Gamma_{y}$, and Γ, were calculated based on the entire warped waveform, as described above. B2B-BPFs and B2B-BPVs were calculated for each intervention. 

### 2.9. Statistics and Linear Models

To determine the effect between pregnancy progression and the introduced B2B-BPF metrics, i.e., $\Gamma_{x}$, $\Gamma_{y},$ and Γ, linear mixed-effects models (LMMs) were employed. These models were designed to assess the connection between WOG groups 21–23, 24–25, 26–27, and 28–30 and corresponding changes in B2B-BPF. To exclude potential interaction effects of the breathing and orthostasis exercises, the initial analysis focused solely on resting intervention I1. Different volunteer compositions per timepoint, repeated measures, and the different anthropometric attributes, i.e., height, weight, and age, were considered in the individual models. LMMs were created using Python 3.10 and the statsmodels package (vers. 0.13). To study the effects of WOG, age, weight, and height on variability or fluctuation, LMMs were generated as (read: fluctuation (fluc) is modeled as a function of wog, age, weight, and height):

fluc~wog+age+weight+height.

To compensate for age, weight, height, and patient distribution per group, LMMs were created while accounting for random variation in the intercepts across different individual levels:

fluc~age+height+weight+(1|individuum_id).

Analysis for the impact of interventions was performed for each WOG group as:

fluc~intervention+age+height+weight.

Linear regression analysis and all other statistical tests were performed using the scipy stats package (vers. 1.7). 

## 3. Results

### 3.1. Relation between Pregnancy Progression and B2B-BP Fluctuation

**Figure 4:**
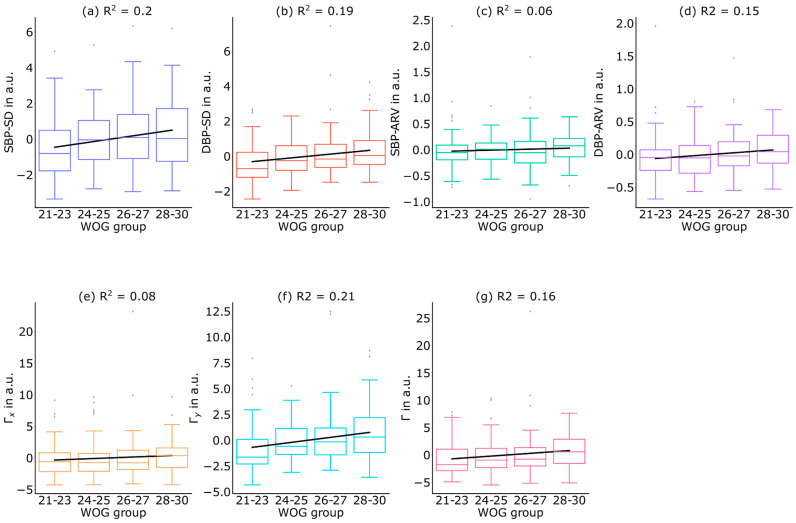
Blood pressure variability and fluctuations between different WOGs. B2B-BP variability parameters, i.e., (**a**) SBP-SD, (**b**) DBP-SD, (**c**) SBP-ARV, and (**d**) DBP-ARV were compared to the fluctuation parameters, i.e., (**e**) $\Gamma_{x}$, (**f**) $\Gamma_{y}$, and (**g**) Γ in regard to BP changes between different WOGs (A). Γy fluctuation values are shown as boxplots (A). The linear trajectories represent the applied linear regression model. Since the composition of volunteers was different between the WOGs, a mixed-effects model was created to regress out height, weight, and age effects. Statistical performance of the LMMs for B2B-BPF and B2B-BPV metrics is shown in Table 2. In addition, Table 3 provides the statistical testing results of the linear regression analysis.

To determine the effect between pregnancy progression and the B2B-BPF metrics $\Gamma_{x},\Gamma_{y}$ and Γ, LMMs were employed, as described in Section 2.9. The generated LMMs revealed a significant impact of pregnancy progression on B2B-BPF metrics $\Gamma_{y}$ and Γ but not $\Gamma_{x}$ (Table 2). Specifically, $\Gamma_{y}$ was significantly increased in WOG 26–27 and WOG 28–30 (*p* < 0.01, *p* < 0.01), and Γ was significantly increased in WOG 28–30 (*p* < 0.05) compared to WOG 21–23. The effects of age, height, and weight differences between the WOG groups were all found to be non-significant (*p* > 0.05, all *p*-values are shown in Table 2), meaning that changes in the women’s physiological attributes are not associated with statistically significant changes in B2B-BPF. B2P-BPF exhibited a consistent increase throughout the progression of pregnancy, as evidenced by the rising coefficients observed in the LMM analysis (Table 2), which demonstrated an upward trend between successive WOGs (Figure 4). For the statistical characterization of this observation, we performed linear regression analysis between B2B-BPF and WOG progression. For noise reduction, WOG group-specific differences in women’s height, weight, and age were eliminated by applying LLMs considering these woman-specific attributes as random effects. Linear regression analysis was performed on the resulting residuals and revealed positive regression slope values for $\Gamma_{x}$, $\Gamma_{y},$ and Γ (Table 3). The goodness of the fit between the linear regression line and the observed B2B-BPF, described by the coefficient of determination $R^{2}$, revealed $R^{2}(\Gamma_{x})$= 0.084, $R^{2}(\Gamma_{y})$ = 0.211, and $R^{2}(\Gamma )$ = 0.156 (Figure 4).

### 3.2. Impact of Exercises on B2B-BP Fluctuations with Regard to WOG

**Figure 5:**
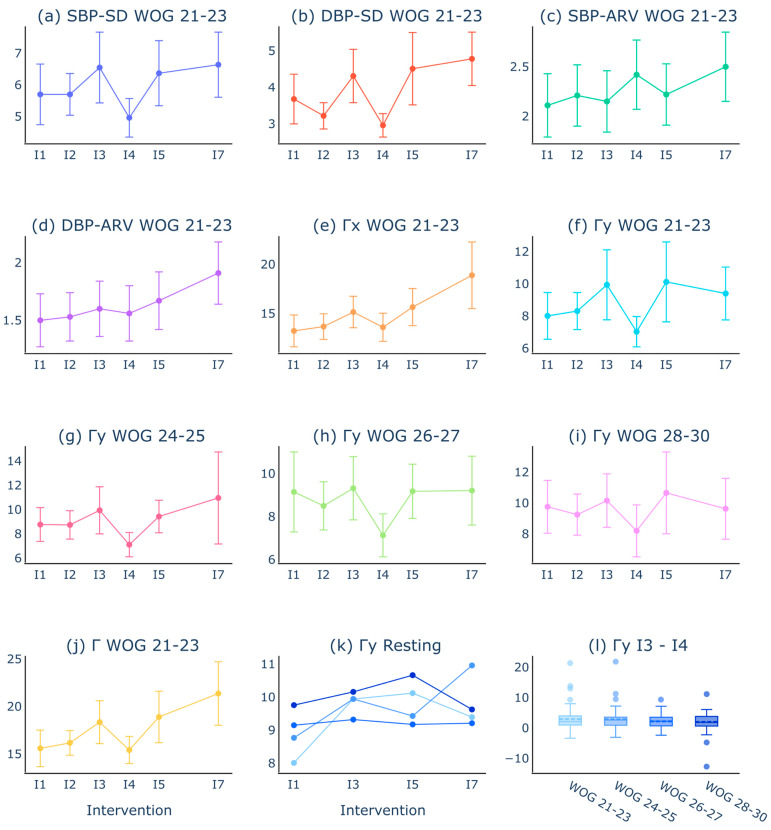
Representative variability and fluctuation mean values compared between interventions. As described in Table 1b, B2B-BP recordings were accessed while the volunteers performed resting (I1, I3, I5, and I7), slow-breathing (I2), fast-breathing (I4), or orthostasis (I6) exercise. (**a**–**j**) show representative B2B-BP changes between the interventions and WOGs. (**k**) Result of the linear regression approach to model B2B-BP changes between the resting interventions. (**l**) Differences between the values from I3 and I4 compared between different WOGs. The detailed statistical performance of LMMs comparing B2B-BPF and B2B-BPV between the resting interventions is shown in Table 4. Table 5 shows the statistical results for LMMs comparing B2B-BPF and B2B-BPV from the fast-breathing intervention to the subsequent resting intervention.

Subsequently, we assessed B2B-BP differences between the breathing and resting exercises with respect to WOGs. BP recordings from intervention I6 were excluded due to high noise caused by the movement of the volunteers from supine to orthostasis. Representative traces of mean B2B-BP values for each exercise are shown in Figure 5a–j.

The analysis of B2B-BPF between the intervals revealed two major findings: (1) B2B-BPF increases from one resting intervention to another, and (2) there is a characteristic decrease in B2B-BPF in the fast-breathing intervention I4. While an interaction model of intervention and WOG group would have only created comparisons to a fixed reference level (intercept), e.g., I1 of WOG 21–23, an LMM for each WOG specifically enabled the comparison of I3, I5, and I7 to I1 of the respective WOG. Table 4 summarizes the outcomes of this approach and reveals significant (*p* < 0.05) differences between all resting interventions I3, I5, and I7, compared to I1 within WOG 21–23, WOG 24–25, and WOG 28–30 for $\Gamma_{x}$ and Γ. Moreover, the B2B-BPF in intervention I7 was significantly different from I1 in all WOGs for Γx and Γ. Γy revealed significant differences in B2B-BPF between all resting interventions I3, I5, and I7, compared to I1 within WOG 21–23 and no significant differences in B2B-BPF in WOG 26–27 or WOG 28–30.

Focusing on the observed B2B-BP value decrease within fast-breathing intervention I4, we performed the same mixed linear model regression approach and adjusted the interventions of interest to resting intervention I3, fast-breathing intervention I4, and resting intervention I5 (Table 5). The statistical analysis revealed significantly decreased B2B-BP values in fast-breathing I4 for all WOG groups for $\Gamma_{y}$ and Γ. However, in $\Gamma_{x}$, I4 was significantly decreased in WOG 21–23, WO 24–25, and WOG 28–30 but not in WOG 26–27. B2B-BP values in resting intervention I5 were not significantly different from resting intervention I3 except for $\Gamma_{x}$ and Γ in WOG 26–27 and WOG 28–30 (Table 5). Since the significant effect of decreased B2B-BP values during fast-breathing intervention was conserved between the WOGs in general, we asked whether the relative difference between I3 and I4 was affected by WOG progression. Therefore, fluctuation parameters from resting intervention I3 and fast-breathing intervention I4 were subtracted for each volunteer and WOG individually and grouped as before into WOG 21–23, WOG 24–25, WOG 26–27, and WOG 28–30, as shown for the fluctuation metric $\Gamma_{y}$ in Figure 5l. To statistically prove the effect of WOG progression on the relation of B2B-BP changes between I3 and I4, we created LMMs for each parameter (Table S1). None of the *p*-values was smaller than 0.05, indicating that WOG progression does not significantly affect B2B-BP differences between resting intervention I3 and fast-breathing intervention I4.

### 3.3. Comparison of B2B-BP Fluctuations and Conventional Variability Measures

As described in Section 2.8, B2B-BPVs, i.e., standard deviation (SD) and average real variability (ARV), were calculated based on the warped systolic indexes (SI) and diastolic indexes (DI). Comparing B2B-BPVs from B2B-BP recordings that were accessed during resting intervention I1 of the FACE study (considered as the baseline) with B2B-BPV values, reported at the baseline before the cold pressure test, we found that the means of the extracted B2B-BPVs were within a similar range as those reported under the baseline in the literature (Table 6). Simultaneous to the B2B-BPF analysis (Section 3.1 and Section 3.2), we compared the relation between pregnancy progression and B2B-BP variability as well as the impact of exercises on B2B-BP variability with regard to WOG. In I1 (baseline) of the FACE study data, the variability measures SBP-SD and DBP-SD were significantly increased in WOG 26–27 (*p* < 0.05) and WOG 28–30 (*p* < 0.05), and DBP-ARV was significantly increased in WOG 28–30 (*p* < 0.05) compared to WOG 21–23, while SBP-ARV did not reveal any significant difference. An investigation of the exercise-induced differences revealed significantly increased variability values between I1, I5, and I7 in B2B-BP data from WOG 21–23 for DBP-SD, SBP-SD, DPB-ARV, and SBP-ARV. Except for SBP-ARV, B2B-BPV was also significantly increased in WOG 21–23 compared to I1. In WOG 24–25, significant B2B-BPV differences were found between I1 and I7 for SBP-SD, DBP-SD, SBP-ARV, and DBP-ARV. In WOG groups 26–27 and 28–30, the analysis did not reveal significant differences between the resting interventions in terms of SBP-SD and DBP-SD. For SBP-ARV and DBP-ARV, B2B-BP variability in I7 remained significantly different in groups WOG 26–27 and WOG 28–30. Considering the observed decrease in B2B-BPF within fast-breathing intervention I4, a significant decrease can be also found within the B2B-BPV metrics SBP-SD and DBP-SD throughout all WOG groups. SBP-ARV was significantly increased in all WOG groups, while DBP-ARV did not reveal significant differences between I3 and I4 in any WOG (Table 5)

## 4. Discussion

In this study, we introduced a new processing pipeline and fluctuation parameters for B2B-BP signals and applied them to weekly acquired non-invasive B2B-BP signals from pregnant women between WOG 21 and 30 from the FACE study [15]. Then, we introduced ELZA, an algorithm to characterize and annotate B2B BP signals more robustly compared to existing annotation tools (Figure 2). B2B-BP changes were extracted with the adopted i2DSW algorithm, which allowed specific feature extraction, such as diastolic (DBP) and systolic (SBP) blood pressure values, as well as entire beat morphology analysis. Validation of the algorithm with artificial signals of different noise levels (Figure 3) was performed to demonstrate the abstinence of technical artifacts. Our novel processing pipeline can be used in different ambulant monitoring approaches and contributes to current developments of new measures of cardiovascular disease (CVD) risk.

There is growing interest in B2B-BP analysis as a key measure of cardiovascular disease (CVD) risk and early-stage disease predictor in pregnancy [21] since recent studies show that 5–10% of all pregnancies [22] affected by hypertension is the most common medical disorder. Furthermore, hypertensive disorders of pregnancy (HDP) are considered the leading cause of maternal and fetal morbidity [22,23]. To reduce future HDP risk in patients, the identification of early-stage disease predictors is crucial. Our study results might contribute towards achieving this goal by providing information about B2B-BP changes within healthy pregnant controls. 

The calculated variability values DBP-SD, DBP-ARV, SBP-SD, and SBP-ARV from the volunteer-specific B2B-BP signals from the FACE study were within the same range of B2B-BP variability as data from the literature [10] (Table 6). This finding indicates comparability between the outcomes produced by our newly implemented processing pipeline and the existing literature data. However, there are notable differences between the volunteers from [10,15]. In [10], data were obtained from male (51/80) and female (29/80) volunteers with a mean age of 25.2 ± 2.4 years, while the BP signals analyzed in this paper were acquired from 44 pregnant women with a mean age of 30 ± 5.4 years.

While conventional measures focus solely on single characteristic points, such as DBP or SBP, we have introduced a fluctuation metric, similar to its equivalent in ECG signal processing [12], to exploit all information from the continuous BP beat signal. Fluctuations can be calculated separately along the x-coordinates ($\Gamma_{x}$), the y-coordinates ($\Gamma_{y}$), or a combination of x- and y-coordinates (Γ). LLM analysis, followed by linear regression modeling, revealed significant increases in $\Gamma_{y}$ between WOG 21–23 and WOG 26–27 as well as WOG 21–23 and WOG 28–30. Since the conventional measures DBP-SD and SBP-SD reveal the same behavior and Γ and DBP-ARV reveal additional significantly increased fluctuation and variability, these results show no contradictions in their physiological meaning. As both variability and fluctuation reveal a significant increase in B2B-BP with increasing WOG, this observation implies a genuine physiological effect rather than a technical or methodological artifact. 

Eventually, we analyzed B2B-BP changes between the performed exercises and identified increasing B2B-BP changes between the resting interventions as well as a significant B2B-BP decrease during the fast-breathing intervention. While all variability and fluctuation metrics revealed a significant difference between I1 and I3, I5, and I7 in WOG 21–23, only SBP-ARV, DBP-ARV, $\Gamma_{x},$ and Γ revealed this significance until WOG 28–30. Similar to our initial relation between pregnancy progression and B2B-BP fluctuation analysis, SBP-SD, DBP-SD, and $\Gamma_{y}$ revealed the same outcome. Overall, these observations show that the significant differences between resting interventions transform into non-significant differences with increasing WOG progression, but not equally, for all B2B-BP parameters. Speculating on whether the B2B-BP value increase in the resting interventions was driven by breathing and orthostasis remains challenging to ascertain, primarily due to the fact that all volunteers performed the breathing exercises and thus, a comparison to a control group is not feasible. 

Finally, the B2B-BP decrease in fast-breathing intervention I4 was significant in all WOG groups and parameters (except DBP-ARV), and the relative decrease was not significantly affected by WOG progression, thus suggesting a genuine physiological effect of fast breathing on B2B-BP changes. While this study focused on the methodological aspects of introducing i2DSW and the fluctuation metric in non-invasive blood pressure measurements, future research in physiological and pathophysiological-oriented studies should prioritize increasing the sample size for enhanced statistical power, accounting for physiological variability.

Taken together, these results suggest a proof of concept for the newly introduced fluctuation metric compared to the conventional state-of-the-art metrics. We demonstrated and validated the robustness of the proposed analysis pipeline to interference signals due to noise as well as the sensitivity to physiological changes in healthy pregnant women during increasing gestational age, agreeing with previous findings from the literature. The introduction of the fluctuation parameter $\Gamma_{x}$ enables the analysis of changes in time resolution, which, in our opinion, had not yet been investigated in B2B-BP research but can now become included in upcoming studies with clinical data. Finally, fluctuation analysis allows one to assess changes along the entire beat morphology rather than individual points, which might be a strong advantage in further analysis applications. 

## 5. Conclusions

Our work contributes a new processing pipeline for B2B-BP changes in cBP signals. The pipeline compromises the ELZA beat-detector, i2DSW, and amplitude and time-specific fluctuation calculation, which was already approved in ECG signal analysis and is now available for BP analysis too. In principle, all types of cBP signals can be analyzed with this pipeline, which might become interesting in ambulant monitoring approaches. As shown frequently in the literature, B2B-BP changes contain precise information about cardiovascular circuits finally equipping our method with high potential in detecting early diagnostic markers in future research by novel non-invasive biosignal sensing techniques. Here, we have demonstrated how the analysis pipeline could aid in the development of new measures for predicting cardiovascular disease (CVD) risk and early-stage disease during pregnancy. We have also presented benchmarks in healthy women that can serve as a reference for comparison with pathological conditions.

## Figures and Tables

**Table 1:** Dataset details summarizing (a) the number (n) of volunteer-specific datasets with the mean ± standard deviation of women’s heights, weights, and ages. In (b), the intervention task and duration that women performed per WOG are shown. In total, 241 datasets were available.

(a) Women Meta Data. Mean ± Standard Deviation				
**WOG**	**n**	**Height in cm**	**Age in years**	**Weight in kg**
21	13	166.5 ± 6.6	32.0 ± 5.9	79.2 ± 11.4
22	19	166.3 ± 7.0	30.8 ± 5.1	71.6 ± 11.6
23	33	166.8 ± 7.5	32.5 ± 5.9	72.9 ± 12.8
24	27	167.0 ± 7.8	31.2 ± 5.8	73.4 ± 14.2
25	32	166.3 ± 8.0	31.8 ± 5.8	73.5 ± 13.6
26	28	166.4 ± 6.9	32.1 ± 5.2	74.9 ± 11.7
27	36	166.5 ± 7.3	31.3 ± 5.6	75.0 ± 14.9
28	27	167.5 ± 6.9	31.8 ± 5.8	74.6 ± 16.7
29	24	166.2 ± 7.5	30.4 ± 5.7	73.0 ± 12.3
30	2	162.5 ± 2.1	25.0 ± 5.7	78.5 ± 14.8
	241	166.2 ± 6.8	30.9 ± 5.7	74.7 ± 13.4
(b) Protocol Details				
**Intervention**		**Task**	**Duration in min**	**Breathing Rate in BPM**
I1		Resting	10	
I2		Paced Breathing	5	8
I3		Resting	5	
I4		Paced Breathing	5	20
I5		Resting	5	
I6		Stand-Up	1	
I7		Resting	5	

WOG = week of gestation and n = amount of datasets.

**Table 2:** LMM results for each investigated B2B-BP parameter. LMMs were generated considering the impact of WOG, age, weight, and height on the respective B2B-BP metric. Coefficients represent the difference between the respective WOG and the intercept while all other variables are held constant. The *p*-value was calculated based on the z-score.

Parameter			Intercept	WOG 24–25	WOG 26–27	WOG 28–30	Age	Weight	Height
B2B-BPV	SBP-SD	Coeff	−0.751	0.595	0.7	1.02	0.004	−0.001	0.001
		*p*-val	0.78	0.058	0.021	0.002	0.832	0.937	0.967
	DBP-SD	Coeff	−0.468	0.266	0.536	0.619	0.002	−0.001	0.001
		*p*-val	0.808	0.23	0.014	0.007	0.864	0.913	0.959
	SBP-ARV	Coeff	−0.033	0.007	0.003	0.069	0.000	−0.000	0.000
		*p*-val	0.953	0.909	0.961	0.302	0.923	0.997	0.996
	DBP-ARV	Coeff	−0.073	−0.005	0.07	0.124	0.001	−0.000	0.000
		*p*-val	0.885	0.939	0.222	0.04	0.872	0.936	0.97
B2B-BPF	Γ_x_	Coeff	−0.444	0.151	0.278	0.774	0.004	−0.000	0.000
		*p*-val	0.928	0.793	0.622	0.189	0.914	0.981	0.989
	Γ_y_	Coeff	−1.125	0.852	1.163	1.491	0.006	−0.001	0.001
		*p*-val	0.773	0.058	0.008	0.001	0.834	0.924	0.964
	Γ	Coeff	−1.041	0.568	0.877	1.592	0.007	−0.001	0.001
		*p*-val	0.852	0.384	0.165	0.019	0.876	0.965	0.977

WOG = week of gestation, B2B-BPV = beat-to-beat blood pressure variability, B2B-BPF = beat-to-beat blood pressure fluctuations, ARV = average real variability, SD = standard deviation, SBP = systolic blood pressure, DBP = diastolic blood pressure, Γ_x_ = fluctuation in time, Γ_y_ = fluctuation in amplitude, and Γ = combined fluctuation in time and amplitude.

**Table 3:** B2B-BP changes between WOGs modeled with linear regression. For each parameter, the mean residual values per WOG group are represented, as well as the linear regression-identified slope, the coefficient of determination, R^2^, and the linear regression’s *p*-value which represents the probability of the relationship between the WOG group and the variability/fluctuation values.

Parameter		WOG 21–23	WOG 24–25	WOG 26–27	WOG 28–30	Slope	R^2^	*p*-Value
B2B-BPV	SBP-SD	−0.554	0.04	0.145	0.461	0.319	0.199	0.002
	DBP-SD	−0.342	−0.077	0.192	0.273	0.215	0.191	0.003
	SBP-ARV	−0.018	−0.01	−0.015	0.051	0.019	0.059	0.362
	DBP-ARV	−0.045	−0.049	0.025	0.079	0.044	0.148	0.021
B2B-BPF	Γ_y_	−0.843	0.008	0.318	0.641	0.485	0.211	0.001
	Γ_x_	−0.279	−0.13	−0.002	0.489	0.237	0.084	0.196
	Γ	−0.719	−0.153	0.156	0.864	0.502	0.156	0.016

WOG = week of gestation, B2B-BPV = beat-to-beat blood pressure variability, B2B-BPF = beat-to-beat blood pressure fluctuations, ARV = average real variability, SD = standard deviation, SBP = systolic blood pressure, DBP = diastolic blood pressure, Γ_x_ = fluctuation in time, Γ_y_ = fluctuation in amplitude, Γ = combined fluctuation in time and amplitude, and R^2^ = coefficient of determination.

**Table 4:** Comparison of B2B-BP differences between the resting interventions. LMMs were designed for each parameter and WOG group to analyze the effect of the intervention on the respective B2B-BP measure. Here, we display the coefficients and *p*-values of the respective LLM.

Parameter			WOG 21–23		WOG 24–25		WOG 26–27		WOG 28–30	
			Coeff	*p*-Value	Coeff	*p*-Value	Coeff	*p*-Value	Coeff	*p*-Value
B2B-BPV	SBP-SD	Intercept	14.78	<0.05	7.19	0.39	4.83	0.36	8.47	0.21
		I3	0.86	<0.05	0.09	0.81	−0.34	0.28	−0.34	0.31
		I5	0.68	0.03	−0.34	0.37	−0.54	0.08	−0.34	0.3
		I7	0.84	0.01	1.27	<0.05	0.05	0.87	−0.11	0.76
	DBP-SD	Intercept	8.98	0.03	3.07	0.52	0.4	0.92	6.07	0.21
		I3	0.63	0.01	0.25	0.34	−0.31	0.17	−0.21	0.38
		I5	0.83	<0.05	0.2	0.44	−0.23	0.29	−0.04	0.88
		I7	1.02	<0.05	1.27	<0.05	0.14	0.56	0.14	0.56
	SBP-ARV	Intercept	3.6	0.09	1.04	0.59	−0.17	0.93	3.11	0.2
		I3	0.04	0.58	0.03	0.71	−0.1	0.11	−0.05	0.63
		I5	0.11	0.09	0.08	0.23	−0.08	0.23	−0.04	0.71
		I7	0.37	<0.05	0.44	<0.05	0.22	<0.05	0.45	<0.05
	DBP-ARV	Intercept	1.91	0.22	−0.46	0.73	−0.32	0.84	1.87	0.33
		I3	0.1	0.09	0.09	0.13	−0.08	0.22	−0.04	0.66
		I5	0.17	<0.05	0.1	0.11	−0.01	0.88	<0.05	0.99
		I7	0.39	<0.05	0.46	<0.05	0.25	<0.05	0.34	0
B2B-BPF	Γx	Intercept	17.69	0.02	4.55	0.69	11.11	0.59	12.47	0.28
		I3	1.93	0.01	3.15	<0.05	1.58	0.29	2.3	<0.05
		I5	2.43	<0.05	3.37	<0.05	3.84	0.01	4.08	<0.05
		I7	5.66	<0.05	6.79	<0.05	6.58	<0.05	5.16	<0.05
	Γy	Intercept	26.62	0.03	15.28	0.25	4.43	0.6	4.24	0.75
		I3	1.93	<0.05	1.18	0.07	0.17	0.7	0.4	0.48
		I5	2.11	<0.05	0.66	0.31	0.03	0.96	0.91	0.11
		I7	1.19	0.02	2.14	<0.05	0.04	0.93	−0.13	0.83
	Γ	Intercept	30.73	0.01	14.29	0.36	11.74	0.59	12.87	0.34
		I3	2.76	<0.05	3.38	<0.05	1.37	0.35	2.09	0.02
		I5	3.3	<0.05	3.2	<0.05	3.36	0.02	4.07	<0.05
		I7	5.69	<0.05	6.86	<0.05	5.93	<0.05	4.41	<0.05

WOG = week of gestation, B2B-BPV = beat-to-beat blood pressure variability, B2B-BPF = beat-to-beat blood pressure fluctuations, ARV = average real variability, SD = standard deviation, SBP = systolic blood pressure, DBP = diastolic blood pressure, Γ_x_ = fluctuation in time, Γ_y_ = fluctuation in amplitude, Γ = combined fluctuation in time and amplitude, Coeff = linear mixed model fixed effect coefficient.

**Table 5:** Effect of fast-breathing intervention on B2B-BP. LMMs were designed for each parameter and WOG group to analyze the effect of fast-breathing intervention in comparison to resting interventions I3 and I5 on the respective B2B-BP measure. Here, we display the coefficients and *p*-values of the respective model.

			WOG 21–23		WOG 24–25		WOG 26–27		WOG 28–30	
			Coeff	*p*-Value	Coeff	*p*-Value	Coeff	*p*-Value	Coeff	*p*-Value
B2B-BPV	SBP-SD	Intercept	12.1	0.03	3.98	0.54	10.41	0.04	9.32	0.13
		I4	−1.6	<0.05	−1.39	<0.05	−0.91	<0.05	−1.05	<0.05
		I5	−0.18	0.48	−0.43	0.2	−0.2	0.35	−0.01	0.98
	BDP-SD	Intercept	8.36	0.06	0.91	0.77	3.98	0.15	5.15	0.24
		I4	−1.35	<0.05	−1.24	<0.05	−1.05	<0.05	−0.98	<0.05
		I5	0.21	0.29	−0.05	0.79	0.08	0.58	0.17	0.47
	SBP-ARV	Intercept	2.4	0.26	0.23	0.9	0.09	0.96	2.68	0.21
		I4	0.27	<0.05	0.28	<0.05	0.28	<0.05	0.19	0.02
		I5	0.07	0.26	0.06	0.46	0.03	0.66	0.01	0.88
	DBP-ARV	Intercept	1.35	0.37	−0.37	0.77	0.44	0.75	1.62	0.36
		I4	−0.04	0.48	−0.08	0.22	−0.02	0.71	−0.05	0.51
		I5	0.07	0.19	0	0.96	0.07	0.25	0.04	0.65
B2B-BPF	Γx	Intercept	26.78	<0.05	7.57	0.56	16.51	0.25	−0.51	0.97
		I4	−1.55	<0.05	−1.99	<0.05	−1.82	0.1	−1.56	0.04
		I5	0.5	0.27	0.22	0.73	2.26	0.04	1.77	0.02
	Γy	Intercept	25.01	0.05	10.15	0.24	13.36	0.08	4.97	0.7
		I4	−2.91	<0.05	−2.84	<0.05	−2.19	<0.05	−1.96	<0.05
		I5	0.18	0.71	−0.51	0.22	−0.15	0.64	0.5	0.39
	Γ	Intercept	37.93	<0.05	11.49	0.4	20.73	0.19	0.52	0.98
		I4	−2.94	<0.05	−3.22	<0.05	−2.67	0.01	−2.29	0.01
		I5	0.54	0.34	−0.18	0.78	1.99	0.06	1.97	0.02

WOG = week of gestation, B2B-BPV = beat-to-beat blood pressure variability, B2B-BPF = beat-to-beat blood pressure fluctuations, ARV = average real variability, SD = standard deviation, SBP = systolic blood pressure, DBP = diastolic blood pressure, Γ_x_ = fluctuation in time, Γ_y_ = fluctuation in amplitude, Γ = combined fluctuation in time and amplitude, Coeff = linear mixed model fixed effect coefficient.

**Table 6:** B2B-BP variability and fluctuation values from intervention I1 compared between different WOGs and datasets. For each parameter, the mean values per WOG group ± standard deviation are presented.

Parameter (Unit)		Our Results (Intervention 1)				[10]
		WOG 21–23	WOG 24–25	WOG 26–27	WOG 28–30	Baseline
B2B-BPV	SBP-SD (mmHg)	5.7 ± 1.91	6.3 ± 1.77	6.38 ± 2.23	6.78 ± 2.17	5.35 ± 1.28
	DBP-SD (mmHg)	3.68 ± 1.36	3.96 ± 1.17	4.24 ± 1.82	4.38 ± 1.36	3.78 ± 0.85
	SBP-ARV (mmHg)	2.11 ± 0.64	2.13 ± 0.51	2.1 ± 0.65	2.15 ± 0.47	1.68 ± 0.38
	DBP-ARV (mmHg)	1.5 ± 0.47	1.51 ± 0.38	1.58 ± 0.48	1.65 ± 0.38	1.39 ± 0.45
B2B-BPV	Γ_x_ (n.u.)	13.23 ± 3.25	13.4 ± 3.39	13.61 ± 4.08	14.03 ± 2.97	-
	Γ_y_ (n.u.)	8.01 ± 2.89	8.77 ± 2.79	9.15 ± 3.73	9.76 ± 3.42	-
	Γ (n.u.)	15.59 ± 3.85	16.17 ± 3.78	16.56 ± 5.00	17.34 ± 3.45	-

B2B-BPV, beat-to-beat blood pressure variability; B2B-BPF, beat-to-beat blood pressure fluctuations, n.u. = normalized unit, ARV = average real variability, SD = standard deviation, SBP = systolic blood pressure, DBP = diastolic blood pressure, Γ_x_ = fluctuation in time, Γ_y_ = fluctuation in amplitude, Γ = combined fluctuation in time and amplitude.

## Data Availability

The data contain sensitive and potentially identifiable information. They cannot be shared publicly because participants restricted their consent to scientific research. The data are available from the Institutional Data Access of the Institute of Biomedical Engineering (contact via Sekretariat.IBMT@tu-dresden.de) for researchers who meet the criteria for access to confidential data. Please provide a clear hypothesis and study protocol.

## Supplementary Materials

The following supporting information can be downloaded at: https://www.mdpi.com/article/10.3390/s24103151/s1, Figure S1: Distribution of women’s anthropometric attributes; Table S1: Fast-breathing exercise comparison.
