# Supplementary material for: Novel Metric for Non-Invasive Beat-to-Beat Blood Pressure Measurements Demonstrates Physiological Blood Pressure Fluctuations during Pregnancy
Source: Sensors (Basel). 2024 May 15;24(10):3151. doi: 10.3390/s24103151 (PMC11125072; doi:10.3390/s24103151)
Supplement: Supplementary file 1 [file sensors-24-03151-s001.zip › sensors-2960343-supplementary.pdf]

Supplementary Materials

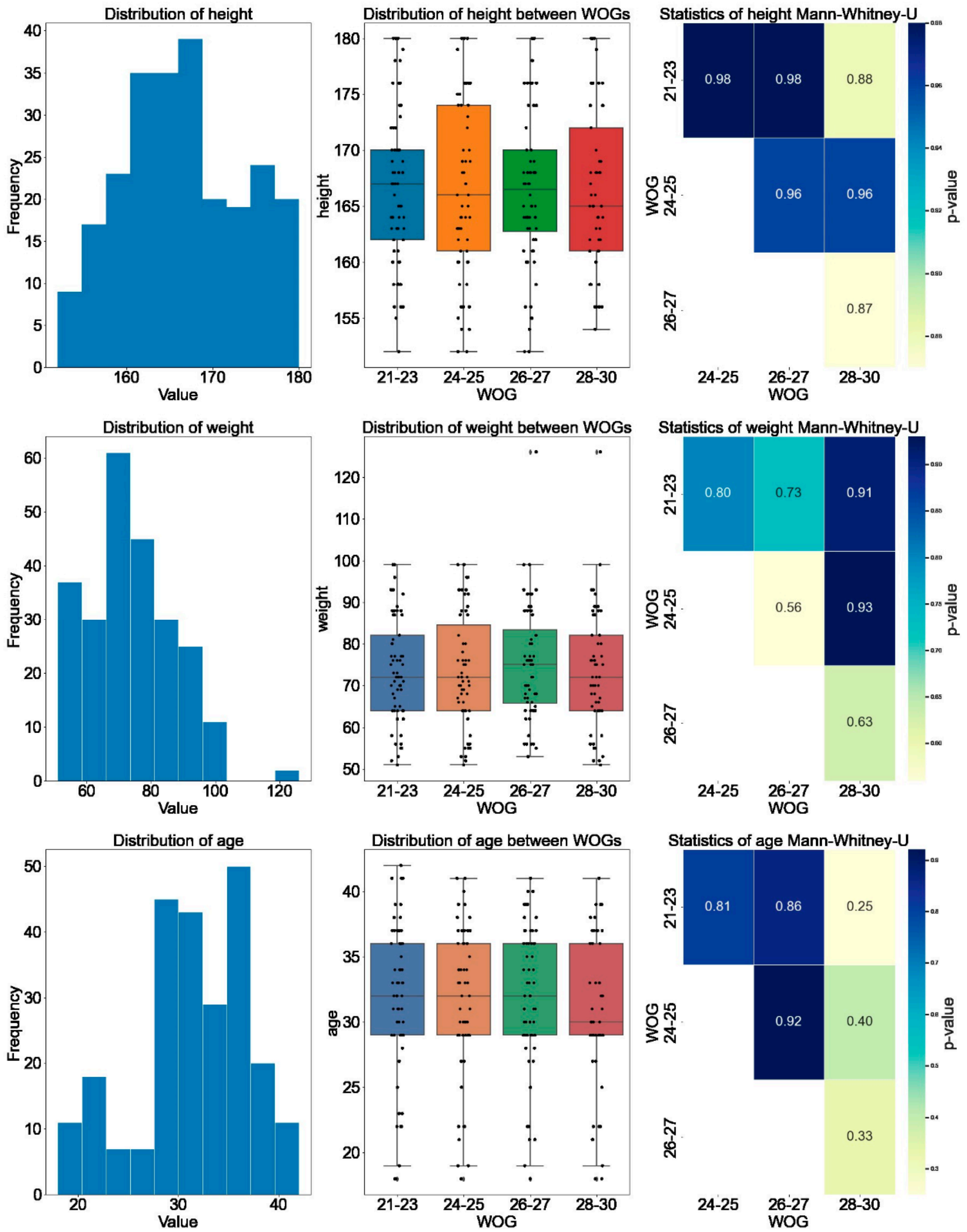

Figure S1: Distribution of women's anthropometric attributes: row 1) height, row 2) weight, and row 3) age. Attributes were analyzed according their frequency (A, D, andG), along their distribution between the aggregated groups WOG 21–23, WOG 24–25, WOG 26–27, and WOG 28–30 (B, E, and H). Mann–Whitney-U tests revealed equal data distribution between the aggregated groups since no p-value was smaller than 0.05.

Table S1: Fast-breathing exercise comparison. Calculated B2B-BPV and B2B-BPF from breathing intervention I4 were subtracted from resting intervention I3. The calculated differences were fitted by of a mixed-effects regression model to estimate the impact of WOG on the decline in intervention 4.

Parameter			I3-I4			
			Intercept	WOG 24-25	WOG 26-27	WOG 28-30
B2B-BPV	SBP-SD	Coeff	1.563	-0.192	-0.513	-0.56
		p-val	<0.001	0.587	0.142	0.124
	DBP-SD	Coeff	1.343	-0.106	-0.22	-0.37
		p-val	<0.001	0.625	0.304	0.098
	SBP-ARV	Coeff	-0.286	0.007	0.024	0.107
		p-val	<0.001	0.927	0.747	0.176
	DBP-ARV	Coeff	0.027	0.046	-0.011	0.054
		p-val	0.692	0.525	0.873	0.475
B2B-BPF	Γ_x	Coeff	1.515	0.551	0.264	0.143
		p-val	0.002	0.406	0.684	0.834
	Γ_y	Coeff	2.949	-0.058	-0.503	-1.025
		p-val	<0.001	0.913	0.337	0.061
	Γ	Coeff	2.897	0.448	-0.15	-0.507
		p-val	<0.001	0.548	0.838	0.509

WOG = week of gestation, B2B-BPV = beat-to-beat blood pressure variability, B2B-BPF = beat-to-beat blood pressure fluctuations, n.u. = normalized unit, ARV = average real variability, SD = standard deviation, SBP = systolic blood pressure, DBP = diastolic blood pressure, Γ_x = fluctuation in time, Γ_y = fluctuation in amplitude, Γ = combined fluctuation in time and amplitude.
